# Strategies used by Iranian nursing students for adjusting to internship: a qualitative study

**DOI:** 10.1186/s12909-023-04441-8

**Published:** 2023-06-20

**Authors:** Hassan Babamohamadi, Naiereh Aghaei, Mohammad Reza Asgari, Nahid Dehghan-Nayeri

**Affiliations:** 1 Nursing care Research Center, Semnan University of Medical Sciences, Semnan, Iran; 2 Department of Nursing, School of Nursing and Midwifery, Semnan University of Medical Sciences, Semnan, Iran; 3 Faculty of Nursing and Midwifery, Mazandaran University of Medical Science, Sari, Iran; 4 Student Research Committee, Semnan University of Medical Sciences, Semnan, Iran; 5 Department of ICU and Nursing Management, School of Nursing and Midwifery, Tehran University of Medical Sciences, Tehran, Iran; 6 Nasibeh School of Nursing and Midwifery, Amir Mazandarani Blvd., Vesal St., Sari, Mazandaran Po Box: 4816715793, Iran

**Keywords:** Nursing students, Internship, Adjustment, Coping strategies, Content analysis

## Abstract

**Background:**

The internship setting is a highly challenging one for nursing students, and working in such an environment requires adjustment. Knowledge of the adjustment strategies used by students enhances the body of nursing knowledge and can help nursing officials adopt appropriate decisions to strengthen the students’ effective adjustment skills and increase the benefits reaped from their internship. The present study was conducted to explore the strategies used by nursing students to adjust to internship.

**Methods:**

A total of 19 senior nursing interns (7 Female, and 12 Male) were selected by purposive sampling with maximum variation from one of the nursing and midwifery schools affiliated to a large metropolitan medical university in northern Iran. Data were collected using audio-taped semi-structured face-to-face interviews over an 18-month period and were carefully transcribed and analyzed using the Graneheim & Lundman qualitative conventional content analysis approach. The researchers analyzed the data in MAXQDA 10 software.

**Results:**

Four main categories and eight subcategories emerged from the data analysis. Main categories include efforts to achieve clinical competency, efforts to be sociable or accepted, self-management and reaction to conflicts.

**Conclusion:**

All the participants attempted to attain adjustment by adopting strategies such as achieving clinical competence, trying to be sociable or accepted, self-management, and reaction to conflicts depending on the conditions of internship. Officials should help nursing students use effective strategies and achieve adjustment.

## Background

Nursing is a combination of science and art. The art of nursing prevails in clinical practice and training, and the science and art of nursing are integrated in this setting [1], to which long hours are dedicated [2]. The clinical learning environment has a major role in nursing students’ opportunities for acquiring professional skills [3].

Iran’s nursing course offers a bachelor’s degree of four years in nursing. The fourth year is dedicated to internship/clerkship, students independently undergo their internship directly supervised by head nurses and clinical personnel, and indirectly by the supervising nursing professors [4, 5]. The internship hours are consistent with those of the nursing personnel [6]. With 21 units equivalent to 1428 h of clinical work, this course is offered in morning, evening and night shifts in different wards [5]. The nursing internship course provides a rich and invaluable experience and an excellent opportunity to obtain clinical skills as well as relating theoretical nursing knowledge to real clinical situations under the guidance and support of experienced supervisors. [7]. As such, this course has a key role in preparing all nursing students for working as professional nurses and reducing the reality shock [8]. The final practical exam is necessary for graduation after the internship is completed [5].

Clinical training and the clinical setting can be stressful for students [9]. Whether an event causes distress or not depends on people’s perception of the situation. A high level of stress and physiological and psychological symptoms are observed among undergraduate nursing students compared to the students of other health disciplines [10], which can ruin the students’ emotional and psychological status and adversely affect their quality of patient care [11].

Since nursing interns have no immunity against clinical stress, adopting effective adjustment strategies to control stress is highly important and can turn a highly stressful situation into a controllable one [12]. Therefore, nursing students must use all the available resources to achieve positive outcomes in dealing with the stress they face [7].

Too many changes occurring simultaneously in life (personal or work-related), albeit positive or negative, may be beyond the individual’s capacity to successfully adjust with the circumstances and might therefore make them ill. It is also possible for the same situation to be disturbing at one point for a given person but exciting for another person or at another time [10]. It is therefore essential to investigate nursing students’ adjustment strategies [13] so that they can be assisted in reinforcing their coping skills and effectively dealing with stressors [14], thus leading to their better adjustment to clinical practice [13].

Adjustment means adjusting oneself so as to deal with a new setting or situation in order to manage, learn, and adapt to that setting or situation, and is a process that occurs over time [13].

Many studies have been conducted on coping and adjustment strategies in nursing students, and their results have shown some of the strategies used by the students, including problem-solving, remaining optimistic, avoidance, and transfer [15–18]. Yet, the results of a review study by Younas (2016) on stress levels and adjustment strategies in nursing students in Asian countries showed that the issue has not been adequately examined due to the methodological constraints found in the reviewed studies and the researchers therefore recommended further studies on the subject [19].

In Iran, students take internship/clerkship courses in the absence of a trainer and only under the supervision of the head nurse and ward personnel, which naturally means that they will be faced with many challenges during this period. It is therefore essential to carry out a profound investigation of adjustment and the sociocultural context affecting its formation [13] and the role of different personal attributes in terms of the perception of stress and the applied strategies, changes in stress levels and the strategies used by nursing students after gaining experience in different clerkship courses and the fact that clerkship courses are taken independently and semi-independently, so that the characteristics of this phenomenon can be examined by a qualitative approach.

This study was conducted on nursing students at Mazandaran University of Medical Sciences to identify the strategies used for adjusting to internship and to then assist them in better adjusting to the internship setting and conditions using positive strategies.

## Methods

### Study design

The present qualitative study was conducted using the Graneheim & Lundman (2004) content analysis approach [20]. Qualitative content analysis was employed because of the goals of the study, i.e., to identify the strategies used by Iranian nursing students for adjusting to internship based on their perceptions and daily experiences. This research approach helps researchers discover and explain people’s perceptions and experiences of everyday phenomena. Although some introduce qualitative content analysis as a textual data analysis technique, it is a systematic research approach involving the formulation of the research question, data collection, and analysis to understand and discover phenomena [21]. It is also a research approach to describe and interpret textual data through a systematic coding process whereby concepts can be grouped into common categories with similar meanings [22, 23]. Qualitative content analysis is divided into conventional (inductive), directed (deductive), and summative methods [22]. Conventional content analysis is an inductive method and, unlike the directed method, it is used to discover participants’ perceptions without involving preconceptions and presuppositions in the research process [21]. In this study, we adopted this approach since we aimed to explore the strategies used by nursing students for adjusting to internship without taking into account mental preconceptions and formulated theories. Qualitative content analysis can be carried out through several methods including Kyngäs et al., 2019; Elo & Kyngäs, 2008; and Graneheim & Lundman,2004 [20, 21, 24]. The present researchers used the method recommended by Graneheim & Lundman (2004) to collect and analyze the data [20]. Used extensively in research articles, this inductive analysis method involves user-friendly, operational, and practical steps.

### Participants

The participants included 19 senior nursing students at one of the nursing and midwifery schools affiliated to a major university of medical sciences in the north of Iran who were willing to take part in the study.

### Data collection

After obtaining permission from the officials of the study setting school, data were collected over an 18-month period from January 2019 to June 2020 through in-depth semi-structured interviews.

The study inclusion criteria were: Being a senior nursing student with no history of working in a hospital. Purposive sampling with maximum variation was used to select the subjects. The purpose of maximum variation is to identify key dimensions of variations and then to find cases that vary from one another as much as possible. In other words, the purpose of maximum variation sampling in qualitative studies is to identify essential features and variable features of a phenomenon among varied contexts [25], and to construct a holistic understanding of the phenomenon [26]. We considered sociodemographic status, gender, marital status, being native or non-native, internship field experience in different wards, and different grade point averages (GPAs) to achieve maximum variation.

To select the key-informant participants, the education deputy of the faculty, the head of the medical-surgical and critical care departments, and a clinical expert of the study setting school were asked to introduce well-spoken students with the ability to properly reflect issues who were a great source of relevant information for assessing the study phenomenon with their extensive experience in internship. The list of interns plus their phone numbers was obtained from the department manager’s office. The necessary arrangements were first made on the phone and the interns were briefed about the study objectives and methods. Finally, the interns’ representative agreed to participate and was selected as the first participant.

Once the interns’ experiences were obtained from different wards under supervision of various personnel and professors, students of the 7th and 8th semester from three consecutive academic years were also recruited. Moreover, the researcher needed to revisit the study setting to further clarify and resolve some ambiguities in the interviews. Some interviews were conducted in mid-semester, some in between two semesters, and some at the end of the semester, and according to the study objectives, students’ experiences were used. It is noteworthy that students were asked to describe their experiences from the commencement of the internship course until the interview time.

After obtaining participants’ consent to take part in the study, the time and place of the interviews were arranged with them. Due to participants’ preference, the interviews were held in a private and comfortable room in the study setting school.

Examples of the interview questions included “Describe a day in the process of internship”, “What problems did you face during your internship?”, and “What did you do to resolve these problems?”, and then, based on the experiences described, probing questions were asked, such as “Could you expand on that?”, or “What do you mean by that?” All the interviews were audio-taped with prior knowledge of the participants.

The first researcher, who had six years of nursing teaching and clinical work experience and was trained in qualitative studies, conducted and transcribed the interviews. Only this first researcher knew participants’ names, and the other researchers only received the interview texts with no names.

The process of data collection continued until data saturation, i.e. when no further new concepts were added to the study after two consecutive interviews, which, in this study, occurred after 16 interviews. Three further interviews were conducted to ensure that data saturation was actually reached.

Five students underwent a second round of interviews to clarify any ambiguities in their interviews or confirm their interview findings. A total of 24 interviews were held with 19 students (19 interviews in the first round and five in the second). The average duration of the interviews was 80.88 min, with a range from 27 to 120 min.

### Data analysis

Data were analyzed using the Graneheim & Lundman (2004) qualitative content analysis approach to extract meaning units, condensed meaning units, codes, subcategories, categories, and themes [20]. A qualitative content analysis approach is used to objectively interpret the contents of textual data and generate knowledge and understanding of the study phenomenon [27], which is usually used for nursing research and education [20].

The interviews were carefully audio-taped and transcribed after they were held, and each text was read several times to achieve a general understanding of them. Then, all the texts were read line by line, and meaning units were identified, condensed, and encoded. Next, the codes were classified into subcategories based on their similarities and differences. The similar subcategories formed the categories, and the theme (i.e. the hidden meaning of the text) thus emerged.

The results were discussed and assessed by all the four researchers. The researchers analyzed the data by using MAXQDA 10 software.

### Study rigor and trustworthiness

The rigor of the study was assessed using Lincoln and Guba’s criteria. To assess qualitative studies, Lincoln and Guba suggested credibility, dependability, confirmability, transferability, and authenticity criteria [28]. These criteria were achieved through such measures as member check (allowing participants to review interviews to confirm or reject certain items), assessment of the encoding by the research team and external check by individuals conversant in research methodology, obtaining written informed consent from all participants, clarification of the study method, rich description of data for a clear understanding of the study process, sampling with maximum variation, and prolonged engagement. Prolonged engagement with the participants helped the first author to gain their trust and obtain a better understanding of the study fields [29].

It is worth noting that all participants studied in the same school and had the same internship conditions. The internship was held in different medical centers, all affiliated with the same university. Although medical centers had different facilities depending on whether they were general or specialized centers, efforts were made to help all students use their clinical experiences in all centers rotational clinical units during their entire internship.

## Results

The participants included 19 senior nursing students who were willing to take part in the study (Table 1).

**Table 1:** Demographic characteristics of the participants

Characteristics	N
**Gender**	
*Female* *Male*	7 12
**Age (years)**	21–25
**Marital status**	
*Single* *Married*	16 3
**Native**	
*Yes* *No*	10 9
GPA*	15-18.40

* Grade point average (GPA)

Four main categories and eight subcategories emerged from the data analysis including efforts to achieve clinical competency, efforts to be sociable or accepted, self-management and reaction to conflicts (Table 2).

**Table 2:** The main categories and subcategories of strategies used by Iranian nursing students for adjusting to internship

Main categories	Subcategories
Effort to achieve clinical competence	Accountability
	Enhancing academic and professional skills
Effort to be sociable or accepted	Trust building
	Seeking support
Self-management	Surrendering
	Foresight and efforts to control the internship conditions and stress
Reaction to conflict	Audacity
	Avoidance

### Effort to achieve clinical competence

All participants tried to adjust to the internship conditions through accountability and enhancing their academic and professional skills.

#### Accountability

All participants tried to be accountable through commitment to work and adherence to the internship rules.

Due to fear of making mistakes when providing care and loneliness because of their practice independent from a teacher in the ward, they tried their best to observe the principles of performing sterile procedures according to what they had learnt and took utmost care in performing the procedures correctly. If not confident in their own knowledge and performance, they asked for the personnel’s help and referred the patient’s problems to the nurse.

Regarding commitment to work, a student explained: “*I try to do my work properly, accept my mistakes and do the right thing*” (P10, male, semester 7).

The participants tried to observe the principles and regulations of the internship. A student who ardently adhered to the regulations pointed out: “*I tried to come and go on time. I asked for the head nurse’s permission whenever I wanted to go somewhere*” (P3, male, semester 8).

#### Enhancing academic and professional skills

Almost all participants made an effort to enhance their academic and professional skills by developing their specialized knowledge and improving their clinical skills.

Due to little competence, the personnel’s lack of confidence in the students’ practical competence, and worrying about care-taking, the participants tried various ways to enhance their specialized knowledge, such as reading textbooks, asking questions of the supervising professors and personnel who cooperated with them, self-learning motivation to deal with new and ambiguous cases, searching available resources, and reviewing the patients’ records.

A participant who was trying to improve his knowledge when placed in the job said: “*Seeing that someone’s life depended on my performance made me try to add to my knowledge a bit*” (P12, male, semester 7).

The students tried to improve their professional skills by independently or non-independently performing the activities in their job description, asking for help when they failed in performing a certain procedure, cooperating with the personnel and seeking duties to get done.

As for improving clinical skills, a participant who was very willing to perform clinical work stated: “*We arranged with the person in charge of the shift that we insert the catheter for any new patient who comes in and send their tests ourselves*” (P9, female, semester 8).

### Effort to be sociable or accepted

In an effort to adjust to internship conditions, participants tried to be accepted by personnel and patients by gaining their trust and support.

#### Trust building

Due to the students’ independent presence in the ward and the personnel’s unfamiliarly with them, the participants tried to gain the personnel’s trust and the trust of the patients and their companions.

When the personnel were unhappy with a student’s performance, some participants tried to gain back their trust by explaining the reason behind their actions or by doing the right work. Furthermore, they tried to gain the personnel’s trust by performing the ward duties under the supervision of the head nurse and meeting their expectations, doing work for the personnel who cooperated well with them, and praising their performance and showing respect to them. One participant argued: “*I worked a lot more with the personnel who cooperated with me and learned more from them*” (P16, female, semester 7).

The students talked with the patients and their companions in order to make them cooperate better and performed the procedure with the patients’ consent. P2, female, semester 8, said: “*When I saw that the IV line was one I could confidently take, I tried to talk to the patient and gain his trust*”.

#### Seeking support

Most participants sought others’ support by raising their issues with the personnel or with the superiors in charge and the senior personnel.

Because of the difficulty of internship and the incompatibility of the assigned tasks with the students’ responsibilities, the participants discussed the ward problems with the personnel by protesting or asking for their help to resolve any issues. Participant 2, female, semester 8, stated: “*Sometimes, I haven’t been able to take a patient’s IV line, and have called Mr. X and said that I can’t take the patient’s IV line, so please come and help me”*.

The participants referred their internship problems to their supervising professors and the group manager. P14, male, semester 7, explained: *“I informed one of the professors that the personnel assign mostly general service duties to me and I don’t do any particular nursing work; they wouldn’t allow me to*”.

### Self-management

Students tried to adjust to internship by controlling internship conditions, foresight and surrendering.

#### Surrendering

Due to the difficulty and problems of internship, all participants had been forced to surrender to improper internship conditions by using a variety of strategies such as coming to terms with adversities, silence and indifference toward the problems.

All the participants somehow surrendered and accepted the conditions by trying to meet the personnel’s work expectations and complying with the ward routine. Participant 13, male, semester 7, said: “*To avoid any conflicts and disappointments, I would not say anything, and said to myself that it was no problem, that I am a nursing student right now, and will accept the things they want from me*”.

Most participants opted for silence in the face of adversities in their internship or when they felt there was no solution to their problem. P14, male, semester 7, discussed surrendering to his poor internship conditions and said: “*Since my professor had said that I would be asked to retake the course, I feared that the personnel would tell him something that would get me failed, so I didn’t say anything*”.

Some participants did not care about the inappropriate reactions of the personnel, the patient, and other internship problems and became apathetic.

#### Foresight and efforts to control the internship conditions and stress

Due to the stress of internship, the participants controlled the internship conditions by trying to understand the conditions of the workplace, being conservative, and self-regulation.

They tried to learn the ward conditions before beginning their internship. They got to know the ward routine by carefully examining the ward in the first few days of the internship and managed the job-related problems and accepted professional nursing problems due to the absence of a trainer in the ward.

Discussing the workplace conditions, a student revealed: “*Before entering a ward, we ask things of the previous groups, or if we are entering as the first group, we ask the students from the previous semesters about how the ward is and we try our best to enter the ward knowing some of its background*” (P15, female, semester 8).

The students were cautious about providing care and assessed the patients’ conditions or informed the nurses if a patient was not willing to cooperate or if they themselves did not feel capable of the duty.

When required, the students attempted to control the situation so that it would not get worse. About self-regulation, a student who had been reproached by the head nurse for causing problems for a patient pointed out: “*I was hurt, since the head nurse was talking loudly to me in the presence of ten other people, and tried so hard to control myself and not get into an argument*” (P19, male, semester 7).

### Reaction to conflicts

About certain issues and problems during internship, participants audaciously expressed their objections to personnel and refused to carry out their duties.

#### Audacity

Because of the personnel’s unreasonable behaviors and the officials’ lack of support for students, some participants disrupted work by working to rules and daring feedbacks of objections.

Whenever the students discussed their issues with the personnel with their supervising professors and the professors took the personnel’s side, they then refused to do the things the personnel asked them to do. Participant 18, female, semester 8, explained: “*For instance, when they asked us to do something, we did it with indifference, didn’t care if it was done properly or not, we just did things to have them done*”.

Participant 8, male, semester 8, who had excellent nursing knowledge and skills, discussed his reaction to the personnel who reproached the students for their lack of skills and said: “*The personnel think that we ought to know everything by semester 6, and if we don’t know something yet, they ask, ‘You are in semester seven, semester eight, how are you going to work with us in a while?’ And we reacted to them and said, ‘As if you all already know everything yourselves!”*

#### Avoidance

Participants refused to perform clinical tasks by avoiding personnel or refusing to carry out their orders when no attention was paid to them, their questions were not answered by personnel, or expected to do things beyond their job description.

One student discussed how he avoided the personnel when he did not receive the answer to his question from them and said: “*Once, when the personnel were talking to each other, I told them that I needed to administer a drug and asked them, ‘What should I do?’ They ignored me completely, as if I didn’t exist at all, and started talking [to each other] again. So, I left the meds there and told them to do it themselves. And never touched it again*” (P11, male, semester 7).

Some participants did not follow the personnel’s style of work and refused to do things beyond their job description. About disobeying the personnel’s orders and refusing their non-clinical requests, one student said: “*A head nurse in this ward had asked me to take photocopies of something and I refused*” (P7, male, semester 8).

## Discussion

The present study results showed that in adjusting to internship conditions, nursing students used a combination of strategies including efforts to gain clinical competence, to be sociable or accepted, self-management and reaction to conflicts.

According to the researchers, all the participants used all the four noted strategies because of their exposure to a range of internship conditions.

The results showed that “efforts to gain clinical competence” was an important and dominant strategy used by all participants in adjusting to internship conditions. In agreement with the present findings, the results of one study showed that the development of professional competence was one of the strategies used by the students to actively deal with the stresses of the clinical setting [30]. In another study, some students eagerly sought to practice as a professional nurse, and following a bad experience, they reformed their behavior [9].

The present findings agree with the results of the cited studies in terms of the types of strategies adopted. Given the students’ independent presence and the absence of a full time academic support in the internship, and the need to acquire readiness for the future work environment, the students gained motivation to improve their academic and clinical skills with the assistance of the personnel, supervising professors, patients and peers.

Another important and prevailing strategy used by participants to adjust to internship conditions was the effort to be sociable or accepted. In agreement with the present findings, the results obtained by Rafati et al. (2017b) revealed participants’ use of communication skills to control stressful interactions and gain the trust of the patients and their families [30]. Friendly rapport with the nurses was one of the strategies used by the students in the study by Bam et al. (2014), who also mainly used social support as a coping strategy [9].

Nonetheless, in disagreement with the present findings, in one study, the least-used strategy was social support seeking [31]. Meanwhile, in another study, one of the methods commonly used by the participants was sharing problems with friends [10].

However, the innovative part of the present study was seeking support from superiors and personnel and sharing problems with them, so that by gaining trust and solving problems at the roots, they can be better accepted by the clinical/personnel and official groups.

Self-management was another strategy used by almost all participants in adjusting to the internship conditions that was carried out with foresight and efforts to control internship stress and conditions, and surrendering. In agreement with the present findings, the results obtained by Rafati et al. (2017b) revealed that when participants were unable to control stressful situations and could not find a solution to their problem, they used the surrendering strategy [30].

In other studies, students mostly used problem-solving for stress minimization [9, 10, 15–18]. Quoting Chan et al., Alsaqri (2017) argued that nursing students who have previously had various learning experiences use problem-solving skills to deal with the stresses of clinical training [17]. The use of the problem-solving strategy in the cited studies is consistent with the present findings in some respects.

Nonetheless, in disagreement with the present findings, remaining optimistic was the most commonly-used strategy in some studies [9, 10, 15–18]. In another study, the most frequently-used adjustment strategy was showing self-confidence and an optimistic approach [31]. The students who were supervised by clinical trainers in a group reported less use of the problem-solving approach compared to students who were individually supervised by a clinical mentor [32]. In another study, problem-solving and remaining optimistic were the least-used adjustment strategies [33].

What makes the present study unique with respect to the above studies was the use of self-management through surrendering in the form of coming to terms with adversities, silence and indifference toward internship problems, foresight and effort to control internship stress and conditions, and also not using optimistic strategies.

According to the researchers, the disagreement between the present study and the above studies in the frequency and type of strategies used can be attributed to the lack of adequate learning opportunities for developing problem-solving skills in the studies whose participants have been selected from lower semesters of the program.

Development of problem-solving skills following different learning opportunities in the course of the nursing program in the present study, the methodological differences [16], and the use of a questionnaire to assess coping behaviors in the cited studies, which may have limited the students’ choice of strategies, and working on students from various academic semesters could have affected the adopted strategies and led to the differences in coping style and behavior.

Also in the present study, the lack of full-time presence of a trainer in the internship and the students’ autonomy were effective in adopting an adjustment strategy and could be a reason for their choice of the problem-solving strategy.

The reason for the use of the above strategy by almost all participants in the present study was the selection of senior nursing students, as they had a greater understanding of matters by this level. By acquiring clinical experience, the students’ view toward issues changes, and as they gradually become more involved in the issues of clinical work during their internship/clerkship (which is a period of transition from being a student to becoming nursing personnel), they try to use strategies that are more effective and also enable them to better control and manage the existing conditions.

In the present study, depending on circumstances, almost all participants did not comply with internship conditions through avoidance, rejection, audacity and expressing problems. In a number of other studies, too, the students used the avoidance strategy [11, 15–18, 32, 33].

In contrast to the present findings, in one study, the strategy least used by the students to cope with stress was the exchange of words in anger with the nurses and the other medical personnel [9]. According to the researchers, the students in this study used the avoidance strategy because they had gained greater knowledge about the internship goals and also more experience in their work. Also, the personnel’s lack of knowledge about the philosophy behind the assignment of students to hospital wards made the students react negatively to the assignment of non-clinical tasks to them. Other reasons included the absence of a trainer in the internship, feeling a lack of support, and the students’ negative perceptions about the tasks assigned to them, the personnel’s indifference toward the students’ education and learning, the lack of understanding for the students’ circumstances and expecting the students to perform any task in the ward, the absence of the required context for implementing the new curriculum, the assignment of a student to the ward and the interns expecting the personnel to treat them like other personnel as they were already working and performing procedures independently in the ward.

Unlike in the present study, using the transfer approach (watching TV, movies, physical exercise, and the like) was the main strategy used by the students in some studies to minimize stress [15, 16].

However, the present study students did not use the transfer strategy because they had already experienced its long-term ineffectiveness to cope with stress in their previous clinical training courses [16].

In contrast to the present findings, mental distraction, realism, positive thinking, stress relief by performing favorite activities, consuming herbal and chemical medications, reducing physiological symptoms, release of emotions [30], talking with one’s parents, sharing problems with friends [10], gaining the family’s moral support [9], and praying were the strategies used by the students to deal with the stresses of clinical settings in other studies. Moreover, self-blame, changes in sleep pattern, avoidance of others, changes in eating habits, blaming others, avoidance of friends, family, and activities, postponing jobs, drinking alcohol, and anger were unhealthy coping methods used by the students [10].

According to the researchers, the reason for this difference can be the selection of a range of participants from the second to the eighth semesters in the cited studies. Students in lower semesters used any method for reducing their stress regardless of its effects because they were unfamiliar with the conditions and had poor experience in handling problems; however, since the interns in the present study already had some experience from their previous semesters at school, they used more solid methods for adjustment to the internship conditions.

Despite participants’ rich experiences of their adopted strategies for adjustment to internship/clerkship, the results have limited generalizability to students from other schools. The results were checked with a number of non-participating students, who confirmed the consistency of the results and their recommendations enhanced the transferability of the findings.

### Strengths and weaknesses

The study limitations included the unwillingness of some interns to take part in the interviews and having the interviews recorded for fear of its adverse effects on their internship and academic progress. The reviewed studies were mostly quantitative [10, 15, 16, 18, 19], or a combination of freshman to senior students were recruited [11, 15, 18, 19]. An innovation of the present study was the recruitment of only senior students who were doing their internship in the absence of a full-time trainer.

### Implications for practice

The present study results can make nursing authorities aware of the strategies used by the internship students, so that they can attempt to solve problems by assessing the root causes of these strategies, especially negative ones, and help students use appropriate strategies for better adjustment to internship conditions.

### Recommendations

Given the absence of the clinical context needed for the implementation of a new internship curriculum (presence of students without the trainer in the internship), further research is recommended to better understand students’ strategies to adjust to internship over several consecutive courses.

## Conclusion

In dealing with the internship conditions, all participants depending on the cause, tried to acquire the required clinical competency, become sociably accepted, control the situation based on the self-management, or avoid surrendering through disobedience and reaction to conflicts.

Given the present findings, officials are recommended to adopt measures to reform the conditions causing adversities in internship/clerkship and thus help students in strengthening their appropriate adjustment strategies, correcting inappropriate strategies, and accomplishing better adjustment for the students’ optimal use of their internship opportunity.

## Data Availability

The datasets used and/or analyzed during the current study available from the corresponding author on reasonable request.
